# Supplementary material for: Mapping the evolution of fertility support policies in China: A content and instrumental analysis
Source: PLoS One. 2025 Oct 9;20(10):e0332137. doi: 10.1371/journal.pone.0332137 (PMC12510515; doi:10.1371/journal.pone.0332137)
Supplement: S1 Appendix — (ZIP) [file pone.0332137.s001.zip › S1 Appendix. 226 original policy documents/168-中华人民共和国人口与计划生育法.doc]

中华人民共和国人口与计划生育法

**发布部门：** [全国人大常委会](https://www.pkulaw.com/law/chl?Aggs.IssueDepartment=201) [C:\email\index](../../../../C:/email/index)

**发文字号：**中华人民共和国主席令第63号

**发布日期：**2001.12.29

**实施日期：**2002.09.01

**时效性：** [已被修改](https://www.pkulaw.com/law/chl?Aggs.TimelinessDic=03)

**效力级别：** [法律](https://www.pkulaw.com/law/chl?Aggs.EffectivenessDic=XA0101)

**法规类别：** [人口计划管理](https://www.pkulaw.com/law/chl?Aggs.Category=09701)

**修改依据：**[全国人民代表大会常务委员会关于修改《中华人民共和国人口与计划生育法》的决定(2015)](javascript:TMP(261789,0,'chl'))

中华人民共和国主席令
（第六十三号）

　　《中华人民共和国人口与计划生育法》已由中华人民共和国第九届全国人民代表大会常务委员会第二十五次会议于２００１年１２月２９日通过，现予公布，自２００２年９月１日起施行。

中华人民共和国主席　江泽民
２００１年１２月２９日

中华人民共和国人口与计划生育法
（２００１年１２月２９日第九届全国人民代表大会常务委员会第二十五次会议通过）
目录

　　第一章　总则
　　第二章　人口发展规划的制定与实施
　　第三章　生育调节
　　第四章　奖励与社会保障
　　第五章　计划生育技术服务
　　第六章　法律责任
　　第七章　附则

第一章　总则

　　第一条　为了实现人口与经济、社会、资源、环境的协调发展，推行计划生育，维护公民的合法权益，促进家庭幸福、民族繁荣与社会进步，根据[宪法](https://www.pkulaw.com/chl/a3b96c9f520bcc3bbdfb.html#class=alink)，制定本法。

　　第二条　我国是人口众多的国家，实行计划生育是国家的基本国策。

　　国家采取综合措施，控制人口数量，提高人口素质。

　　国家依靠宣传教育、科学技术进步、综合服务、建立健全奖励和社会保障制度，开展人口与计划生育工作。

　　第三条　开展人口与计划生育工作，应当与增加妇女受教育和就业机会、增进妇女健康、提高妇女地位相结合。

　　第四条　各级人民政府及其工作人员在推行计划生育工作中应当严格依法行政，文明执法，不得侵犯公民的合法权益。

　　计划生育行政部门及其工作人员依法执行公务受法律保护。

　　第五条　国务院领导全国的人口与计划生育工作。

　　地方各级人民政府领导本行政区域内的人口与计划生育工作。

　　第六条　国务院计划生育行政部门负责全国计划生育工作和与计划生育有关的人口工作。

　　县级以上地方各级人民政府计划生育行政部门负责本行政区域内的计划生育工作和与计划生育有关的人口工作。

　　县级以上各级人民政府其他有关部门在各自的职责范围内，负责有关的人口与计划生育工作。

　　第七条　工会、共产主义青年团、妇女联合会及计划生育协会等社会团体、企业事业组织和公民应当协助人民政府开展人口与计划生育工作。

　　第八条　国家对在人口与计划生育工作中作出显著成绩的组织和个人，给予奖励。

第二章　人口发展规划的制定与实施

　　第九条　国务院编制人口发展规划，并将其纳入国民经济和社会发展计划。

　　县级以上地方各级人民政府根据全国人口发展规划以及上一级人民政府人口发展规划，结合当地实际情况编制本行政区域的人口发展规划，并将其纳入国民经济和社会发展计划。

　　第十条　县级以上各级人民政府根据人口发展规划，制定人口与计划生育实施方案并组织实施。

　　县级以上各级人民政府计划生育行政部门负责实施人口与计划生育实施方案的日常工作。

　　乡、民族乡、镇的人民政府和城市街道办事处负责本管辖区域内的人口与计划生育工作，贯彻落实人口与计划生育实施方案。

　　第十一条　人口与计划生育实施方案应当规定控制人口数量，加强母婴保健，提高人口素质的措施。

　　第十二条　村民委员会、居民委员会应当依法做好计划生育工作。

　　机关、部队、社会团体、企业事业组织应当做好本单位的计划生育工作。

　　第十三条　计划生育、教育、科技、文化、卫生、民政、新闻出版、广播电视等部门应当组织开展人口与计划生育宣传教育。

　　大众传媒负有开展人口与计划生育的社会公益性宣传的义务。

　　学校应当在学生中，以符合受教育者特征的适当方式，有计划地开展生理卫生教育、青春期教育或者性健康教育。

　　第十四条　流动人口的计划生育工作由其户籍所在地和现居住地的人民政府共同负责管理，以现居住地为主。

　　第十五条　国家根据国民经济和社会发展状况逐步提高人口与计划生育经费投入的总体水平。各级人民政府应当保障人口与计划生育工作必要的经费。

　　各级人民政府应当对贫困地区、少数民族地区开展人口与计划生育工作给予重点扶持。

　　国家鼓励社会团体、企业事业组织和个人为人口与计划生育工作提供捐助。

　　任何单位和个人不得截留、克扣、挪用人口与计划生育工作费用。

　　第十六条　国家鼓励开展人口与计划生育领域的科学研究和对外交流与合作。

第三章　生育调节

　　第十七条　公民有生育的权利，也有依法实行计划生育的义务，夫妻双方在实行计划生育中负有共同的责任。

　　第十八条　国家稳定现行生育政策，鼓励公民晚婚晚育，提倡一对夫妻生育一个子女；符合法律、法规规定条件的，可以要求安排生育第二个子女。具体办法由省、自治区、直辖市人民代表大会或者其常务委员会规定。

　　少数民族也要实行计划生育，具体办法由省、自治区、直辖市人民代表大会或者其常务委员会规定。

　　第十九条　实行计划生育，以避孕为主。

　　国家创造条件，保障公民知情选择安全、有效、适宜的避孕节育措施。实施避孕节育手术，应当保证受术者的安全。

　　第二十条　育龄夫妻应当自觉落实计划生育避孕节育措施，接受计划生育技术服务指导。

　　预防和减少非意愿妊娠。

　　第二十一条　实行计划生育的育龄夫妻免费享受国家规定的基本项目的计划生育技术服务。

　　前款规定所需经费，按照国家有关规定列入财政预算或者由社会保险予以保障。

　　第二十二条　禁止歧视、虐待生育女婴的妇女和不育的妇女。

　　禁止歧视、虐待、遗弃女婴。

第四章　奖励与社会保障

　　第二十三条　国家对实行计划生育的夫妻，按照规定给予奖励。

　　第二十四条　国家建立、健全基本养老保险、基本医疗保险、生育保险和社会福利等社会保障制度，促进计划生育。

　　国家鼓励保险公司举办有利于计划生育的保险项目。

　　有条件的地方可以根据政府引导、农民自愿的原则，在农村实行多种形式的养老保障办法。

　　第二十五条　公民晚婚晚育，可以获得延长婚假、生育假的奖励或者其他福利待遇。

　　第二十六条　妇女怀孕、生育和哺乳期间，按照国家有关规定享受特殊劳动保护并可以获得帮助和补偿。

　　公民实行计划生育手术，享受国家规定的休假；地方人民政府可以给予奖励。

　　第二十七条　自愿终身只生育一个子女的夫妻，国家发给《独生子女父母光荣证》。

　　获得《独生子女父母光荣证》的夫妻，按照国家和省、自治区、直辖市有关规定享受独生子女父母奖励。

　　法律、法规或者规章规定给予终身只生育一个子女的夫妻奖励的措施中由其所在单位落实的，有关单位应当执行。

　　独生子女发生意外伤残、死亡，其父母不再生育和收养子女的，地方人民政府应当给予必要的帮助。

　　第二十八条　地方各级人民政府对农村实行计划生育的家庭发展经济，给予资金、技术、培训等方面的支持、优惠；对实行计划生育的贫困家庭，在扶贫贷款、以工代赈、扶贫项目和社会救济等方面给予优先照顾。

　　第二十九条　本章规定的奖励措施，省、自治区、直辖市和较大的市的人民代表大会及其常务委员会或者人民政府可以依据本法和有关法律、行政法规的规定，结合当地实际情况，制定具体实施办法。

第五章　计划生育技术服务

　　第三十条　国家建立婚前保健、孕产期保健制度，防止或者减少出生缺陷，提高出生婴儿健康水平。

　　第三十一条　各级人民政府应当采取措施，保障公民享有计划生育技术服务，提高公民的生殖健康水平。

　　第三十二条　地方各级人民政府应当合理配置、综合利用卫生资源，建立、健全由计划生育技术服务机构和从事计划生育技术服务的医疗、保健机构组成的计划生育技术服务网络，改善技术服务设施和条件，提高技术服务水平。

　　第三十三条　计划生育技术服务机构和从事计划生育技术服务的医疗、保健机构应当在各自的职责范围内，针对育龄人群开展人口与计划生育基础知识宣传教育，对已婚育龄妇女开展孕情检查、随访服务工作，承担计划生育、生殖保健的咨询、指导和技术服务。

　　第三十四条　计划生育技术服务人员应当指导实行计划生育的公民选择安全、有效、适宜的避孕措施。

　　对已生育子女的夫妻，提倡选择长效避孕措施。

　　国家鼓励计划生育新技术、新药具的研究、应用和推广。

　　第三十五条　严禁利用超声技术和其他技术手段进行非医学需要的胎儿性别鉴定；严禁非医学需要的选择性别的人工终止妊娠。

第六章　法律责任

　　第三十六条　违反本法规定，有下列行为之一的，由计划生育行政部门或者卫生行政部门依据职权责令改正，给予警告，没收违法所得；违法所得一万元以上的，处违法所得二倍以上六倍以下的罚款；没有违法所得或者违法所得不足一万元的，处一万元以上三万元以下的罚款；情节严重的，由原发证机关吊销执业证书；构成犯罪的，依法追究刑事责任：

　　（一）非法为他人施行计划生育手术的；

　　（二）利用超声技术和其他技术手段为他人进行非医学需要的胎儿性别鉴定或者选择性别的人工终止妊娠的；

　　（三）实施假节育手术、进行假医学鉴定、出具假计划生育证明的。

　　第三十七条　伪造、变造、买卖计划生育证明，由计划生育行政部门没收违法所得，违法所得五千元以上的，处违法所得二倍以上十倍以下的罚款；没有违法所得或者违法所得不足五千元的，处五千元以上二万元以下的罚款；构成犯罪的，依法追究刑事责任。

　　以不正当手段取得计划生育证明的，由计划生育行政部门取消其计划生育证明；出具证明的单位有过错的，对直接负责的主管人员和其他直接责任人员依法给予行政处分。

　　第三十八条　计划生育技术服务人员违章操作或者延误抢救、诊治，造成严重后果的，依照有关法律、行政法规的规定承担相应的法律责任。

　　第三十九条　国家机关工作人员在计划生育工作中，有下列行为之一，构成犯罪的，依法追究刑事责任；尚不构成犯罪的，依法给予行政处分；有违法所得的，没收违法所得：

　　（一）侵犯公民人身权、财产权和其他合法权益的；

　　（二）滥用职权、玩忽职守、徇私舞弊的；

　　（三）索取、收受贿赂的；

　　（四）截留、克扣、挪用、贪污计划生育经费或者社会抚养费的；

　　（五）虚报、瞒报、伪造、篡改或者拒报人口与计划生育统计数据的。

　　第四十条　违反本法规定，不履行协助计划生育管理义务的，由有关地方人民政府责令改正，并给予通报批评；对直接负责的主管人员和其他直接责任人员依法给予行政处分。

　　第四十一条　不符合本法第十八条规定生育子女的公民，应当依法缴纳社会抚养费。

　　未在规定的期限内足额缴纳应当缴纳的社会抚养费的，自欠缴之日起，按照国家有关规定加收滞纳金；仍不缴纳的，由作出征收决定的计划生育行政部门依法向人民法院申请强制执行。

　　第四十二条　按照本法第四十一条规定缴纳社会抚养费的人员，是国家工作人员的，还应当依法给予行政处分；其他人员还应当由其所在单位或者组织给予纪律处分。

　　第四十三条　拒绝、阻碍计划生育行政部门及其工作人员依法执行公务的，由计划生育行政部门给予批评教育并予以制止；构成违反治安管理行为的，依法给予治安管理处罚；构成犯罪的，依法追究刑事责任。

　　第四十四条　公民、法人或者其他组织认为行政机关在实施计划生育管理过程中侵犯其合法权益，可以依法申请行政复议或者提起行政诉讼。

第七章　附则

　　第四十五条　流动人口计划生育工作的具体管理办法、计划生育技术服务的具体管理办法和社会抚养费的征收管理办法，由国务院制定。

　　第四十六条　中国人民解放军执行本法的具体办法，由中央军事委员会依据本法制定。

　　第四十七条　本法自２００２年９月１日起施行。
